# Role of Vitamin D in Insulin Secretion and Insulin Sensitivity for Glucose Homeostasis

**DOI:** 10.1155/2010/351385

**Published:** 2009-08-19

**Authors:** Jessica A. Alvarez, Ambika Ashraf

**Affiliations:** ^1^Department of Nutrition Sciences, University of Alabama at Birmingham, AL 35233, USA; ^2^Department of Pediatrics, Division of Pediatric Endocrinology and Metabolism, The Children's Hospital, University of Alabama at Birmingham, AL 35233, USA

## Abstract

Vitamin D functions are not limited to skeletal health benefits and may extend to preservation of insulin secretion and insulin sensitivity. This review summarizes the literature related to potential vitamin D influences on glucose homeostasis and insulin sensitivity. Cross-sectional data provide some evidence that circulating 25-hydroxyvitamin D (25(OH)D) is inversely associated with insulin resistance, although direct measurements of insulin sensitivity are required for confirmation. Reported associations with insulin secretion, however, are contradictory. Available prospective studies support a protective influence of high 25(OH)D concentrations on type 2 diabetes mellitus risk. There is a general lack of consistency in vitamin D intervention outcomes on insulin secretion and sensitivity, likely due to differences in subject populations, length of interventions, and forms of vitamin D supplementation. Vitamin D receptor gene polymorphisms and vitamin D interactions with the insulin like growth factor system may further influence glucose homeostasis. The ambiguity of optimal vitamin D dosing regimens and optimal therapeutic concentrations of serum 25(OH)D limit available intervention studies. Future studies, including cross-sectional and prospective, should be performed in populations at high risk for both vitamin D deficiency and type 2 diabetes mellitus. Well-designed, placebo-controlled, randomized intervention studies are required to establish a true protective influence of vitamin D on glucose homeostasis.

## 1. Introduction

Over the last decade, numerous nonskeletal disease associations have been reported with vitamin D deficiency, including type 2 diabetes mellitus (T2DM). Circulating 25-hydroxyvitamin D (25(OH)D) concentrations are considered an indicator of vitamin D status [1, 2]. Compared to healthy controls, subjects with T2DM have been observed to have significantly lower circulating 25(OH)D concentrations [3–5]. Perhaps not coincidently, both vitamin D deficiency and T2DM share the same risk factors, including African-American, Asian, or Hispanic ethnicity, increased adiposity, age, and physical inactivity (which may translate to decreased time spent outdoors or reduced sun exposure) [6, 7]. Seasonal variations in glucose and insulin concentrations have been reported [8], which may correlate with seasonal variations in 25(OH)D concentrations [9]. Although not within the scope of this review, vitamin D has likewise been implicated in the development of type 1 diabetes mellitus due to its modulation of the immune system [10]. T2DM is considered a state of insulin resistance (beta cell compensation) and insulinopenia (beta cell decompensation) and is characterized by progressive deterioration in beta cell function and eventual loss of beta cell mass [11]. The mechanism by which vitamin D deficiency and T2DM are related is not well known.

 We performed a computerized PubMed search of English language original and review articles through March 2009 using the terms “vitamin D”, “insulin”, “insulin sensitivity”, “insulin resistance”, “insulin secretion”, and other related terms. This review provides a comprehensive summary of the literature available relating vitamin D to insulin secretion and sensitivity, including the potential mechanisms mediating the vitamin D-glucose homeostasis relationship; supportive or contradictory cross-sectional, prospective, and intervention studies; as well as potential interactions of vitamin D with the insulin like growth factor (IGF) system and influences of vitamin D receptor (VDR) gene polymorphisms.

## 2. Potential Mechanisms of Effect of Vitamin D on Glucose Homeostasis

Pittas et al. [9] have summarized the biological evidence implicating a potential influence of vitamin D on glucose homeostasis. The inferences for the manifold roles of vitamin D include the presence of specific vitamin D receptors (VDRs) on pancreatic *β*-cells [12], the expression of 1-*α*-hydroxylase enzyme in pancreatic *β*-cells which catalyzes the conversion of 25(OH)D to 1, 25-dihydroxyvitamin D (1,25(OH)_2_D) [13], the presence of a vitamin D response element in the human insulin gene promoter [14], and the presence of VDR in skeletal muscle [15]. In addition, 1,25(OH)_2_Ddirectly activates transcription of the human insulin receptor gene [16], activates peroxisome proliferator activator receptor-*δ* [17], stimulates the expression of insulin receptor, and enhances insulin-mediated glucose transport in vitro [18].

Animal and in vitro studies provide compelling evidence that vitamin D may play a functional role in the preservation of glucose tolerance through its effects on insulin secretion and insulin sensitivity. Vitamin D deficient rabbits and mice present with impaired insulin secretion, and supplementation with vitamin D corrects the defect [19–22]. Mice with mutations in the VDR have impaired insulin secretion and lower glucose tolerance than those with functional receptors [23]. In vitro, 1,25(OH)_2_D induces the biosynthesis of insulin in rat pancreatic islet cells [24], and in another study, inhibited free fatty acid-induced insulin resistance (i.e., improved glucose uptake) in cultured myocytes in a dose-dependent manner. The insulin sensitizing effects were mediated by a reduction in JNK activation [25].

Although the skeletal effects of vitamin D occur via an endocrine mechanism, there may be an autocrine/paracrine role of vitamin D in insulin target tissues. Pancreatic *β*-cells express the vitamin D receptor (VDR) as well as the pivotal enzyme 1*α*-hydroxylase [12, 13]. VDR is also expressed by both human skeletal muscle and adipose tissue [26, 27], which are the main determinants of peripheral insulin sensitivity. These tissues were shown to express the 1*α*-hydroxylase gene in male Wistar rats [28]. Notably, skeletal muscle expression of VDR declines with age [29], as does insulin sensitivity.

Vitamin D deficiency may influence its effects on insulin secretion and sensitivity via its effects on intracellular calcium [9]. Elevated intracellular calcium impairs postreceptor binding insulin action, such as the dephosphorylation of glycogen synthase and of insulin regulatable glucose transporter (GLUT-4) [30, 31]. Vitamin D deficiency results in elevated parathyroid hormone (PTH) [2], which in turn is known to elevate intracellular calcium [31]. Sustained elevations of intracellular calcium may inhibit insulin-target cells from sensing the brisk intracellular calcium fluxes necessary for insulin action, such as glucose transport [32]. Pancreatic *β*-cells also depend on an acute intracellular calcium increase for insulin secretion [33], which may also be attenuated with elevated cytosolic calcium [34]. Another possible mechanism is that elevated intracellular calcium enhances calmodulin binding to insulin receptor substrate-1 (IRS-1), which interferes with insulin-stimulated tyrosine phosphorylation and PI3-kinase activation [35–37]. Indeed, PTH has been shown to be inversely associated with insulin sensitivity [38, 39]. On the other hand, Kamycheva et al. [40] did not find significant differences in insulin or glucose metabolism in subjects with secondary hyperparathyroidism versus controls. Nevertheless, dichotomization based on serum 25(OH)D concentrations appeared to determine differences in insulin sensitivity. It is arguable that the insulin resistance seen in vitamin D deficient subjects is not fully explained by these aforementioned molecular mechanisms alone.

## 3. Cross-Sectional Data

Reports on associations between insulin secretion and 25(OH)D have been inconsistent (Table 1(a)). The differences in this relationship are likely due to differences in subject populations and disparate methods to determine insulin secretion. Boucher et al. [41] reported a significant positive association between 25(OH)D and oral glucose tolerance test- (OGTT-) induced insulin secretion in East London Asians at risk for T2DM, although Orwoll et al. [42] reported no association between 25(OH)D and meal-induced insulin secretion in men with T2DM (mean glycosylated hemoglobin, HbA1c: 11.5 ± 3.5%). It is possible that vitamin D is unable to augment insulin secretion in uncontrolled T2DM subjects who have already exhausted their insulin secretory capacity. Analyses of the National Health and Nutrition Examination Survey 1989–1994 (NHANES III) disclosed that serum 25(OH)D was inversely associated with diabetes risk and measures of insulin resistance despite there is no association between 25(OH)D concentrations and the homeostasis model assessment of *β*-cell function (HOMA-*β*, an index of *β*-cell function derived from fasting insulin and glucose concentrations) [5]. Significant inverse associations have been reported between 25(OH)D and OGTT-induced insulin secretion in elderly Dutch men [4], hyperglycemic clamp-induced insulin response in glucose tolerant subjects of various ethnic backgrounds [43], and hyperglycemic clamp-induced insulin response in Norwegian subjects with secondary hyperparathyroidism [40]. Although an inverse association between 25(OH)D and insulin secretion may seem contradictory to the hypothesis that vitamin D is necessary for *β*-cell synthesis of insulin, subjects with insulin resistance, but not T2DM, often experience compensatory hyperinsulinemia [44]. Thus, inverse statistical associations of vitamin D with insulin secretion may be mediated through vitamin D influences on insulin sensitivity, as shown by Chiu et al. [43].

In general, cross-sectional studies, including large-scale population studies such as NHANES III [5, 45], have shown a significant positive relationship between serum 25(OH)D and measures of insulin sensitivity [4, 5, 40, 43, 45–48] (Table 1(b)). This relationship has been corroborated in a variety of populations including pregnant and pediatric populations [49, 50].

A limitation to the available cross-sectional data is that, with the exception of few studies [40, 43, 51, 52], many studies investigating relationships between 25(OH)D and insulin secretion and sensitivity have used indirect proxy measures, such as fasting insulin, fasting or postchallenge glucose, the homeostasis model assessment of insulin resistance (HOMA-IR), HOMA-*β*, the quantitative insulin-sensitivity index (QUICKI), postchallenge insulin, or HbA1c [4, 5, 42, 46–49, 53], instead of the paragon investigation and the hyperglycemic clamp. The accuracy of proxy measures of insulin sensitivity may vary depending on obesity status or ethnicity [54]. In addition, the majority of studies investigating the association between 25(OH)D and insulin metabolism have used BMI, rather than a direct measure of adiposity as a covariate in analyses [4, 5, 40, 41, 43, 51, 53, 55]. The accuracy of BMI in reflecting adiposity has been questioned, and when studies have used both dual energy X-ray absorptiometry-(DXA-) derived total body fat and BMI in models to predict 25(OH)D, only total body fat emerged as an independent predictor [56–58]. Also, many studies have not accounted for confounders that may be mediating associations between 25(OH)D and insulin sensitivity, such as physical activity and calcium intake [9], each of which has been shown to be significantly associated with insulin sensitivity and may, thus, corrupt data analysis. Furthermore, inherent to the nature of the cross-sectional study design, studies using this design are limited in their ability to infer causation.

Insulin sensitivity may not be influenced by circulating 25(OH)D in some populations. Manco et al. [59] investigated the associations of 25(OH)D with insulin sensitivity (determined with a euglycemic-hyperinsulinemic clamp) in morbidly obese Caucasian women. Serum 25(OH)D was not associated with insulin sensitivity in these subjects either before bariatric surgery or 5 and 10 years post-surgery. Suggesting that the often found low serum 25(OH)D concentrations before and after bariatric surgery do not negatively affect insulin sensitivity. Other anthropometric factors, such as the extreme adiposity prior to surgery and the improved metabolic and lipid profile postsurgery, likely had a greater impact than 25(OH)D on insulin sensitivity. NHANES III data did not show a significant relationship between 25(OH)D and HOMA-IR in African Americans despite there are significant results in Caucasian and Mexican Americans [5], and Alemzadeh et al. [50] reported a significant relationship between serum 25(OH)D and HbA1c in Caucasian but not in African Americans. Similarly, in a meta-analysis of the association between 25(OH)D and T2DM prevalence, Pittas et al. [9] found an OR of 0.36 (95% CI: 0.16–0.08) for the highest concentrations of 25(OH)D compared to the lowest, although this significant OR only appeared after African Americans were excluded from analyses. It is unclear whether disparate ethnicities have different optimal serum concentrations of 25(OH)D, and the relationships of serum 25(OH)D with glucose homeostasis should be examined in African Americans using direct measures of insulin sensitivity and secretion to confirm a nugatory effect of 25(OH)D.

## 4. Prospective Studies

Few studies have examined the predictive value of 25(OH)D on future risk of T2DM [60–62]. Forouhi et al. [61] found baseline 25(OH)D to be inversely associated with fasting glucose, fasting insulin, and HOMA-IR at the 10-year follow-up of the Medical Research Council Ely Prospective Study (European-origin adults), independent of baseline outcome values. Similarly, in the Mini-Finland Health Study, the relative risk for T2DM was 0.60 in subjects with the highest 25(OH)D quartiles (mean 70.9 nmol/L) compared to those in the lowest quartile (mean 22.4 nmol/L, *P* = .01), after adjustment for age, sex, and month of blood draw [60]. This observation, however, was subsequently negated to nonsignificance (*P* = .05–.07) after further adjustments for confounders such as BMI and leisure-time exercise. A pooled, nested case-control analysis of the Mini-Finland Health Study and the Finnish Mobile Clinic Health Examination Survey revealed an 82% reduced risk of T2DM incidence in men with the highest 25(OH)D quartiles (mean 69.11 nmol/L) versus those with the lowest quartiles (mean 22.3 nmol/L, *P* < .001) after adjustment for BMI, physical activity, smoking, and education [62]. A statistically significant reduced risk was not shown in women. Intermediate markers of T2DM risk, such as insulin resistance, were not reported in the latter studies. The role of serum 25(OH)D in predicting the risk for T2DM and insulin resistance in non-Caucasian ethnic populations, such as those with African, Asian, and Indian-descent is worth investigating, as these populations are at high risk for T2DM and low 25(OH)D concentrations.

## 5. Intervention Studies

Table 2(a) summarizes results of available vitamin D intervention studies on insulin secretion. Gedik and Akalin [65] first reported impairment in insulin secretion in 4 relatively healthy subjects presenting with vitamin D deficiency, and their insulin secretion was normalized after 6 months of vitamin D supplementation. Other studies have reported significant improvement in insulin secretion after variable doses and lengths of vitamin D_3_ supplementation in subjects with or at risk for T2DM [41, 66], as did a study using alphacalcidiol as the intervention [67]. Antithetically, studies that have used 1, 25(OH)_2_Dto supplement have not shown significant improvement in insulin secretion [42, 68]. Insofar as the pancreatic *β*-cell is endowed with the ability to produce 1, 25(OH)_2_D by an autocrine mechanism, supplementation with vitamin D_3_ or D_2_ to produce a rise in circulating 25(OH)D may be superior as 25(OH)D serves as the necessary substrate for extra-renal 1-*α*-hydroxylase [90, 91]. Of note, there was a tendency toward a better insulin secretory response in recently diagnosed (within 3 years) T2DM subjects supplemented with 1, 25(OH)_2_D [42], supporting the previous notion that vitamin D supplementation may not be useful once *β*-cells are exhausted. Studies using HOMA-*β*, an indirect index of *β*-cell function derived from fasting values of glucose and insulin, as the insulin secretory outcome, have likewise not shown significant changes in insulin secretion [69, 70]. The majority of available studies, regardless of a positive or negative outcome, are limited by their lack of a randomized, placebo-controlled design [41, 65, 66, 68], and/or nonreporting of serum 25(OH)D to ensure attainment of sufficient vitamin D concentrations [42, 65, 67, 68].

With regards to insulin sensitivity, studies in subjects with chronic renal disease have shown intravenous vitamin D administration to improve insulin sensitivity [92, 93]. Human studies in relatively healthy populations without renal disease have been inconsistent, particularly those using indirect indices of insulin sensitivity (Table 2(b)). Studies using fasting values of glucose and insulin (e.g., HOMA-IR), which primarily reflect hepatic insulin sensitivity, have generally not shown significant improvements in insulin sensitivity after vitamin D intervention [66, 69, 71, 72]. Posthoc analyses, however, from a randomized placebo-controlled trial revealed improved HOMA-IR after 3 years of 700 IU vitamin D_3_ plus 500 mg calcium citrate daily supplementation in Caucasian subjects with impaired fasting glucose, but not normal fasting glucose [73]. This study could not discriminate the relative influences of vitamin D over calcium. Vitamin D supplementation is more likely to influence peripheral insulin sensitivity, as shown by Nagpal et al. [70], whereby 3 doses of 120000 IU vitamin D_3_ fortnightly versus placebo showed significant improvements in a 3-hour OGTT-derived insulin sensitivity index, but not indices derived from fasting values, in Asian-Indian men. Additional studies that have used OGTT-derived indices are limited by relatively small sample sizes, lack of randomized placebo-controlled design, and short-term supplementation [74, 75].

The few studies investigating vitamin D effects on directly measured insulin sensitivity using the euglycemic clamp technique or intravenous glucose tolerance testing (IVGTT) have not shown improvements in insulin sensitivity with supplementation [52, 76, 77] (Table 2(b)). The null effects in 2 studies [52, 76] may, at least in part, be explained by the vitamin D sufficiency of the subject populations. As suggested by Jorde and Figenschau [69], supplementation of vitamin D-sufficient populations may not incur additional glucose regulating effects. In addition, all studies used the active form of vitamin D (1, 25(OH)_2_D) or an analog of this hormone to supplement, and the increase in 25(OH)D was minimal in one study [76] and unable to be evaluated in the others [52, 77]. It is plausible that results would have differed in vitamin D deficient subjects supplemented with cholecalciferol or ergocalciferol to produce a rise in serum 25(OH)D.

There is no universally accepted definition for the optimal serum concentration of 25(OH) D, thus complicating vitamin D supplementation trials. For prevention of rickets or osteomalacia, a serum 25(OH)D concentration of 25 mmol/L (10 ng/mL) is considered sufficient; yet to prevent osteoporosis and maximize calcium absorption a concentration of 75 nmol/L (30 ng/mL) is suggested [1]. Vitamin D deficiency is defined as serum 25(OH)D < 50 nmol/L (<20 ng/mL) [94]. As summarized by Pepper et al. [94], there is also no universally accepted standard regimen for the correction of vitamin D deficiency. It is also unknown what length of intervention or duration of follow-up once vitamin D is replete is required to appreciate the effects of vitamin D on insulin sensitivity and secretion. Further confounding conclusions are that the reported vitamin D intervention studies are heterogeneous in their research design and length, form, and dosage of vitamin D supplementation. Many also lack the demonstration of achieving a therapeutic level of vitamin D. Additionally, the majority of intervention trials have been performed in Caucasian subjects. There is a need for more intervention studies in subjects of non-Caucasian descent.

## 6. Vitamin D Receptor Polymorphisms

Polymorphisms in the VDR gene, namely, *Taq*I*, Bsm*I*, Apa*I, and *Fok*I, have been identified and may influence insulin secretion and sensitivity, although relatively few studies have been conducted and, results have varied (Table 3). Among a cohort of Bangladeshi Asians, the *Apa*I VDR gene polymorphism was associated with insulin secretion index [79]; however reanalysis of this cohort revealed *Taq*I to be the independent predictor of the insulin secretion index [80]. In contrast, the *Bsm*I VDR gene polymorphism was associated with postprandial C-peptide concentrations among Caucasian Hungarians [78]. The *Apa*I and *Bsm*I VDR gene polymorphisms were also shown to be associated with fasting glucose and HOMA-IR, respectively, among a large cohort of Caucasian Americans [83], and *Bsm*I was associated with fasting glucose in a large cohort of Germans [84]. The linkage disequilibrium that exists between the *Taq*I*, Bsm*I and * Apa*I polymorphisms may partly explain the varying results [80, 88]. The *Fok*I VDR polymorphism was also associated with indices of insulin resistance among Polish men [81] and among Caucasian Americans [82], although the studies contradicted each other regarding the specific genotype associated with insulin resistance (FF versus ff).

Despite the support for a VDR gene polymorphism influencing insulin secretion and resistance, case-control studies, in general, have not found statistical differences in VDR gene polymorphism frequencies among subjects with T2DM versus controls [86–89]. Ortlepp et al. [84] did, however, report a higher prevalence of T2DM among German subjects with the BB genotype of the *Bsm*I VDR gene polymorphism compared to those with the bb genotype. The association between VDR gene polymorphisms and insulin secretion and sensitivity should be confirmed using direct methods, such as IVGTT or clamp studies. In addition, these associations should be investigated in African Americans and other non-Caucasian ethnicities.

## 7. Vitamin D and the Insulin-Like Growth Factor (IGF) System

There is some evidence to suggest that vitamin D status may interact with the IGF system to influence glucose homeostasis [61, 64]. Analysis of the 1958 British Birth Cohort revealed a lower risk for metabolic syndrome in subjects with the highest tertiles of both serum 25(OH)D and IGF-1 concentrations, although there was no statistical interaction between 25(OH)D and IGF-1 in any of the individual components of the metabolic syndrome and HbA1c [64]. In a prospective study, Forouhi et al. [61] found an inverse relationship between baseline 25(OH)D and 10-year follow-up fasting and 2-hour glucose only in subjects with baseline IGF binding protein-1 (IGFBP-1) concentrations below the median. An interaction between vitamin D and the IGF axis to influence glucose homeostasis seems conceivable as each has been shown to directly enhance the other [95, 96].

## 8. Summary and Conclusion

There is mechanistic support that vitamin D may influence both insulin secretion and insulin sensitivity and subsequently T2DM incidence. In general, cross-sectional and prospective studies support the role of vitamin D in the prevention of T2DM. Despite the inherent limitations of cross-sectional and prospective study designs, these types of study designs are useful for preliminary research to suggest which specific populations may respond to vitamin D interventions. Future cross-sectional and prospective studies should focus on non-Caucasian ethnicities with a high risk of both T2DM and vitamin D deficiency. Cross-sectional and prospective studies should account for potential confounders of the vitamin D-T2DM relationship, including age, ethnicity, obesity, physical activity, and diet, as well as use direct measures of adiposity, insulin sensitivity, and insulin secretion. Results of vitamin D intervention studies are equivocal; yet many studies are flawed by lack of randomized, placebo-controlled design, use of indirect measures of insulin secretion and sensitivity, small sample size, and inability to show relevant increases in serum 25(OH)D. The lack of protocol for optimal dosing of vitamin D and lack of definition for optimal therapeutic concentrations of serum 25(OH)D further inhibit the application of vitamin D intervention trials. One could also postulate that interactions with the IGF system, VDR gene polymorphisms, or other individual genotypes of subjects might becloud the conflicting conclusions. It is plausible that vitamin D has a role in improving insulin secretion and sensitivity, but may not be effective in insulinopenic situations, or that beyond a certain concentration of vitamin D level, further supplementation of vitamin D sufficient subjects may not be of value in improving glucose homeostasis.

Based on a review of literature, a true, direct link between vitamin D and risk for T2DM has not yet been conclusively established, although several unknowns remain. It is not known what the optimal concentration of vitamin D for glucose homeostasis should be and what duration of follow-up is necessary to appreciate the effects of vitamin D on insulin secretion and sensitivity. It is also unclear if a serum 25(OH)D threshold exists for which vitamin D does not incur additional benefits for glucose homeostasis, if vitamin D influences are ethnic-specific, or if there are different optimal thresholds for different ethnic groups. Large, well-controlled, randomized studies are required to clarify these important unknowns and define the relationship between vitamin D status and glucose homeostasis.

## Figures and Tables

**Table 1:** Reported associations of 25(OH)D with insulin secretion and sensitivity/resistance. To convert nmol/L to ng/mL, divide by 2.496. 25(OH)D: 25-hydroxyvitamin D; OGTT: oral glucose tolerance test; T2DM: type 2 diabetes mellitus; NHANES III: National Health and Nutrition Examination Survey 1988–1994; CA: Caucasian American; AA: African American; MA: Mexican American; HOMA-*β*: homeostasis model assessment of *β*-cell function; HOMA-IR: homeostasis model assessment of insulin resistance; QUICKI: quantitative insulin sensitivity check index; HbA1c: glycosylated hemoglobin; WHR: waist-to-hip ratio; BIA: bioelectrical impedance.

Author, year (ref)	Subjects' characteristics	Mean serum 25(OH)D	Method to determine insulin outcomes	Association of 25(OH)D with insulin outcome	Covariates	Major limitations
			(a) Insulin Secretion			

Boucher 1995 [41]	44 glucose-intolerant East London Asians, mean age 44.9 years, mean BMI: 25.9 kg/m^2^	<27.5 nmol/L	OGTT	Positive association with postchallenge C-peptide and insulin	Age, sex, BMI	No measure of dietary intake or physical activity; indirect measures of adiposity and insulin secretion
Orwoll et al. 1994 [42]	35 adults with T2DM, mean age: 61 years, mean BMI: 29.8 kg/m^2^ (ethnicity not reported)	35 ± 7 nmol/L	Meal challenge	No association with fasting or postchallenge glucose, insulin, C-peptide, or glucagon	—	Indirect measure of insulin secretion; no adjustment for confounders
Scragg et al. 2004 [5]	6228 NHANES III participants (Caucasian, African, and Mexican American), ages ≥ 20 years	79.6 ± 36.8 nmol/L (CA) 49.1 ± 37.5 nmol/L (AA) 66.0 ± 41.5 nmol/L (MA)	Fasting glucose and insulin (HOMA-*β*)	No association with HOMA-*β*	Age, sex, BMI, physical activity, season, HOMA-IR	No measure of dietary intake; indirect measures of adiposity and insulin secretion
Baynes et al. 1997 [4]	142 Dutch men, mean age: 75.7 years	42 ± 29.1 nmol/L	OGTT	Inverse association with postchallenge glucose and insulin	Season, physical activity, BMI, skinfolds, smoking, alcohol, and fish, fat, and oil consumption	No adjustment for calcium consumption; indirect measure of adiposity
Chiu 2004 et al. [43]	126 glucose-tolerant Asian, African, Caucasian, and Mexican American, mean age 26 ± 6 years, mean BMI: 24.7 kg/m^2^	46.9 nmol/L (Asian American), 47.3 nmol/L (AA), 69.4 nmol/L (CA), 50.2 nmol/L (MA)	Hyperglycemic clamp	Inverse association with first- and second-phase insulin response (not significant after adjustment for insulin sensitivity index)	Age, sex, ethnicity, BMI, WHR, blood pressure, season, insulin sensitivity index	No measure of dietary intake or physical activity; indirect measure of adiposity
Kamycheva et al. 2007 [40]	15 Norwegian subjects with secondary hyperparathyroidism, mean age: 62.9 years, mean BMI: 27.1 kg/m^2^; 15 sex-, age-, and BMI-matched controls	58.4 nmol/L (patients) 61.9 nmol/L (controls)	Hyperglycemic clamp	Inverse association with 2nd phase insulin secretion	Sex, age, BMI	No measure of dietary intake or physical activity; indirect measure of adiposity

(b) Insulin sensitivity/resistance						

Scragg et al. 2004 [5]	6228 NHANES IIIparticipants (Caucasian, African, and Mexican American), ages ≥ 20 years	79.6 ± 36.8 nmol/L (CA) 49.1 ± 37.5 nmol/L (AA) 66.0 ± 41.5 mol/L (MA)	Fasting glucose and insulin (HOMA-IR)	Inverse association with HOMA-IR in Caucasian *( * *P* = .058) and Mexican Americans (*P* = .002)	Age, sex, BMI, physical activity, season	No measure of dietary intake; indirect measures of adiposity and insulin sensitivity
Chonchol and Scragg 2007 [45]	14697 NHANES III participants (Caucasian, African, and Mexican American), ages ≥ 20 years	78 ± 58.4 nmol/L (men) 71.6 ± 61.5 nmol/L (women)	Fasting glucose and insulin (HOMA-IR)	Inverse association with fasting insulin and HOMA-IR	Age, sex, ethnicity, BMI	No measure of dietary intake or physical activity; indirect measures of adiposity and insulin sensitivity
Lu et al. 2009 [48]	3262 Chinese adults (Nutrition and Health of Aging Population in China study), ages 50–70 years	40.4 nmol/L	Fasting insulin and glucose (HOMA-IR)	Inverse association with fasting glucose, HbA1c, fasting insulin, and HOMA-IR.	Age, sex, residence, season, education, physical activity, smoking, alcohol, family history, CRP, IL-6, BMI	No measure of dietary intake; indirect measures of adiposity and insulin sensitivity
Gannagé-Yared et al. 2009 [47]	381 Lebanese university students; ages 18–30 years; mean BMI: 23.9 ± 4.1 kg/m^2^	77.4 ± 31.2 nmol/L	Fasting insulin and glucose (HOMA-IR)	Inverse association with fasting glucose, fasting insulin, and HOMA-IR. Only glucose held after adjustment for all confounders.	Sex, BMI, exercise	No measure of dietary intake; indirect measures of adiposity and insulin sensitivity
Clifton-Bligh et al. 2008 [49]	307 pregnant women of various ethnicities, mean age: 32.6 years	53.8 ± 23.9 nmol/L	Fasting insulin and glucose (HOMA-IR)	Inverse association with fasting insulin, glucose, and HOMA-IR but not after adjustment for confounders	Age, BMI, ethnicity	No measure of dietary intake or physical activity; indirect measures of adiposity and insulin sensitivity
Alemzadeh et al. 2008 [49]	127 children (Caucasian, Mexican, and African American), mean age: 13 years, BMI > 95th percentile for age	59.9 ± 23.2 nmol/L	Fasting insulin and glucose (QUICKI), HbA1c	Inverse association with HbA1c, but not after adjustment for confounders	Fat mass (BIA), age, sex, ethnicity, season	No measure of dietary intake or physical activity; indirect measures of adiposity and insulin sensitivity
McGill et al. 2008 [46]	243 adults of various ethnicities (New Zealand), mean age: 47.6 years, mean BMI: 35.4 kg/m^2^	62.2 ± 22.7 nmol/L	Fasting glucose and HbA1c	Inverse association with HbA1c and glucose (negated after removal of 3 outliers)	Sex, age, ethnicity, season, BMI	No measure of dietary intake or physical activity; indirect measures of adiposity and insulin sensitivity
Ford et al. 2005 [53]	8421 NHANESIII participants, ages ≥ 20 years, multiple ethnicities	74 nmol/L (range: 8.7–227.9 nmol/L)	Fasting glucose	Inverse association with fasting glucose	Age, sex, ethnicity, education, smoking, cotinine, cholesterol, abdominal obesity, triglyceridemia, low HDL, high blood pressure	No measure of dietary intake or physical activity; indirect measures of adiposity and insulin sensitivity
Need et al. 2005 [63]	753 postmenopausal Caucasian (Australian) women, mean age: 63 years, mean BMI: 26.5 kg/m^2^	62 ± 24.3 nmol/L	Fasting glucose	Inverse association with fasting glucose	Age, BMI	No measure of dietary intake or physical activity; indirect measures of adiposity and insulin sensitivity
Hyppönen et al. 2008 [64]	6810 British Caucasian adults, age: 45 years, BMI variable	53.8 nmol/L (men) 51.5 nmol/L (women)	HbA1c	Inverse association with HbA1c	Sex, month, IGF-1, physical activity, smoking, alcohol, social class, abdominal obesity, BMI	No measure of dietary intake; indirect measures of adiposity and insulin sensitivity
Baynes et al. 1997 [4]	142 Dutch men, mean age: 75.7 years	42 ± 29.1 nmol/L	OGTT	Inverse association with fasting insulin	—	—
Chiu et al. 2004 [43]	126 glucose-tolerant Asian, African, Caucasian, and Mexican American, mean age 26 ± 6 years, mean BMI: 24.7 kg/m^2^	46.9 nmol/L (Asian American), 47.3 nmol/L (AA), 69.4 nmol/L (CA), 50.2 nmol/L (MA)	OGTT Hyperglycemic clamp	Inverse association with postchallenge glucose; Positive association with insulin sensitivity index	Age, sex, ethnicity, BMI, WHR, blood pressure, season	No measure of dietary intake or physical activity; indirect measure of adiposity
Kamycheva et al. 2007 [40]	15 Norwegian subjects with secondary hyperparathyroidism, mean age: 62.9 years, mean BMI: 27.1 kg/m^2^ and 15 sex-, age-, and BMI-matched controls	58.4 ± 15.3 nmol/L (patients) 61.9 ± 18.5 nmol/L (controls)	Hyperglycemic clamp	Positive association with insulin sensitivity index among all subjects	Sex, age, BMI	No measure of dietary intake or physical activity; indirect measure of adiposity
Lind et al. 1995 [51]	34 Caucasian men, mean age: 63 years	90 ± 19 nmol/L	Euglycemic clamp	Positive association with insulin sensitivity, inverse association with fasting insulin	Age, BMI, WHR, serum creatinine	No measure of dietary intake or physical activity; indirect measure of adiposity
Lind et al. 1989 [52]	10 Danish men with impaired glucose tolerance, ages 60–63 years	107 ± 62 nmol/L	Euglycemic clamp	Positive association with insulin sensitivity	Serum calcium, phosphate, magnesium, and other minerals	No measure of dietary intake or physical activity; no adjustment for adiposity
Manco et al. 2005 [59]	116 Caucasian, morbidly obese (mean BMI: 48.8 kg/m^2^) women before and after bariatric surgery, ages 20–35 years	Pre-surgery: 39.2 ± 22.3 nmol/L, 5 years postsurgery: 27.4 ± 16.4 nmol/L, 10 years postsurgery: 25.1 ± 13.9 nmol/L	Euglycemic clamp	No association with insulin sensitivity before or after bariatric surgery	Triglycerides, fat mass, PTH, serum calcium, phosphorus, HDL, LDL, total cholesterol	No measure of dietary intake or physical activity

**Table 2:** Effects of vitamin D intervention on insulin secretion and sensitivity/resistance. To convert nmol/L to ng/mL, divide by 2.496. 25(OH)D: 25-hydroxyvitamin D; OGTT: oral glucose tolerance test; IVGTT: intravenous glucose tolerance test; T2DM: type 2 diabetes mellitus; 1, 25(OH)_2 _D_3_: 1,25-dihydroxyvitamin D_3_ or calcitriol; HOMA-*β*: homeostasis model assessment of *β*-cell function; NFG: normal fasting glucose; IFG: impaired fasting glucose; HOMA-IR: homeostasis model assessment of insulin resistance; HbA1c: glycosylated hemoglobin.

Author, year (ref)	Subject characteristics	Baseline 25(OH)D	Determinant of insulin/glucose metabolism	Route, dose, and form of vitamin D administration	Length of intervention	Follow-up 25(OH)D	Outcome	Major limitations
(a) Insulin secretion								

Gedik et al. 1986 [65]	4 vitamin D deficient women (Turkey), mean age: 32.7 years, mean BMI: 22.8 kg/m^2^	—	OGTT	Oral, 2000 IU/d cholecalciferol	6 months	—	Increased insulin area and insulinogenic index	Not randomized, placebo-controlled; 25(OH)D not assessed; small sample size
Boucher et al. 1995 [41]	22 glucose-intolerant East London Asians, mean age 44.9 years, mean BMI: 25.9 kg/m^2^)	9.0 ± 4.5 nmol/L	OGTT	Intravenous, 100,000 IU cholecalciferol	Single dose, follow-up 8–12 weeks later	33.7 ± 18.5 nmol/L	Increase in postchallenge insulin and C-peptide	Not randomized, placebo-controlled
Borissova 2003 [66]	10 Bulgarian women with T2DM, mean age: 53.8 years, mean BMI: 30.9 kg/m^2^	35.3 ± 15.1 nmol/L	IVGTT	Oral, 1332 IU cholecalciferol/d	1 month	63.3 ± 31 nmol/L	Increased first-phase insulin secretion	Not randomized, placebo-controlled; small sample size
Inomata et al. 1986 [67]	14 Japanese T2DM subjects, mean age 54.3 years	—	OGTT	2 *μ*g/d alphacalcidiol versus placebo	3 weeks	—	Improved insulin secretion (area under the curve) and reduced free fatty acid concentrations	25(OH)D not reported; small sample size
Zofková and Stolba 1990 [68]	13 vitamin D-sufficient adults, mean age: 33.4 years (ethnicity not reported)	—	IVGTT	Oral, 3 *μ*g/d 1,25(OH)_2_D_3_	4 days	—	No change in insulin secretion	Not randomized, placebo-controlled; 25(OH)D not reported; small sample size
Orwoll et al. 1994 [42]	35 adults with T2DM, mean age: 61 years, mean BMI: 29.8 kg/m^2^ (ethnicity not reported)	35 ± 7 nmol/L	Meal challenge	1 *μ*g/d 1, 25(OH)_2_D versus placebo	4 days	—	No change, but tendency towards better insulin secretion in recently diagnosed subjects (within 3 years)	Postintervention 25(OH)D not assessed
Jorde and Figenschau 2009 [69]	32 Norwegian adults with insulin and metformin-controlled T2DM, ages 21–75, mean BMI: 32.8 kg/m^2^ (treatment), 31.3 kg/m^2^ (placebo)	60 ± 14 nmol/L (treatment) 58.5 ± 21 nmol/L (placebo)	Fasting insulin and glucose (HOMA-*β*) and c-peptide	40,000 IU cholecalciferol/wk versus placebo	6 months	118.3 nmol/L (treatment) 57.2 nmol/L (placebo)	No change in insulin secretion	Sample size insufficient based on power calculations; indirect measure of insulin secretion
Nagpal et al. 2009 [70]	100 Asian Indian, centrally-obese males, age ≥ 35 years, BMI: 26.7 kg/m^2^ (treatment), 26 kg/m (placebo)	36.5 ± 14.6 nmol/L (treatment) 30 ± 12.5 nmol/L (placebo)	Fasting insulin and glucose (HOMA-*β*)	Oral, 3 doses of 120,000 IU cholecalciferol fortnightly versus placebo	6 weeks	71.6 nmol/L (treatment) 30.6 nmol/L (placebo)	No change in insulin secretion	Indirect measure of insulin secretion

(b) Insulin sensitivity								

De Boer et al. 2008 [71]	795–866 postmenopausal women of various ethnicities, ages 50–79 years	43.7 nmol/L (median)	Fasting insulin and glucose (HOMA-IR)	Oral, 400 IU cholecalciferol + 1000 mg calcium versus placebo	6 years	—	No change in fasting glucose, insulin, or HOMA-IR; no change in diabetes risk	Postintervention 25(OH)D not reported; indirect measure of insulin sensitivity
Nilas and Christiansen 1984 [72]	151 Danish postmenopausal women, ages 45–54 years	—	Blood glucose	Oral, 2000 IU/d cholecalciferol + 500 mg/d calcium versus 0.25 *μ*g/d alphacalcidiol + 500 mg/d calcium versus placebo	2 years	—	No change in blood glucose	25(OH)D not reported; indirect measure of insulin sensitivity
Jorde and Figenschau 2009 [69]	32 Norwegian adults with insulin and metformin-controlled T2DM, ages 21–75, mean BMI: 32.8 kg/m^2^ (treatment), 31.3 kg/m^2^ (placebo)	60 ± 14 nmol/L (treatment) 58.5 ± 21 nmol/L (placebo)	Fasting insulin and glucose (HOMA-IR)	40,000 IU cholecalciferol/wk versus placebo	6 months	118.3 nmol/L (treatment) 57.2 nmol/L (placebo)	No change in fasting glucose, insulin, HOMA-IR, or HbA1c	Sample size insufficient based on power calculations; indirect measure of insulin sensitivity
Borissova et al. 2003 [66]	10 Bulgarian women with T2DM, mean age: 53.8 years, mean BMI: 30.9 kg/m^2^	35.3 ± 15.1 nmol/L	Fasting insulin and glucose (HOMA-IR)	Oral, 1332 IU cholecalciferol/d	1 month	63.3 ± 31.0 nmol/L	Nonsignificant decrease in HOMA-IR	Not randomized, placebo-controlled; indirect measure of insulin sensitivity
Pittas et al. 2007 [73]	314 Caucasian American adults, mean age: 71.2 years, mean BMI: 26.7 kg/m^2^	Treatment: 81.4 ± 3.7 nmol/L (NFG), 71.2 ± 5.2 nmol/L (IFG); Placebo: 70.6 ± 2.8 nmol/L (NFG), 81.2 ± 4.7 (IFG)	Fasting insulin and glucose (HOMA-IR)	Oral, 700 IU cholecalciferol + 500 mg calcium versus placebo	3 years	Treatment: 111 nmol/L (NFG), 102.4 nmol/L (IFG); Placebo: 69.7 nmol/L (NFG), 73.4 nmol/L (IFG)	Improved HOMA-IR in subjects with IFG	Indirect measure of insulin sensitivity
Nagpal et al. 2009 [70]	100 Asian Indian, centrally-obese males, age ≥ 35 years, BMI: 26.7 kg/m (treatment), 26 kg/m^2^ (placebo)	36.5 ± 14.6 nmol/L (treatment) 30 ± 12.5 nmol/L (placebo)	OGTT	Oral, 3 doses of 120,000 IU cholecalciferol fortnightly versus placebo	6 weeks	71.6 nmol/L (treatment) 30.6 nmol/L (placebo)	Increased insulin sensitivity (3-hour oral glucose insulin sensitivity index); no change in indices derived from fasting glucose and insulin values	Indirect measure of insulin sensitivity
Tai et al. 2008 [74]	33 primarily Caucasian adults, mean age 55 years, mean BMI: 24.1 kg/m^2^	39.9 ± 8.6 nmol/L	OGTT, fasting insulin and glucose	Oral, 2 doses of 100,000 IU cholecalciferol	1 month (Follow-up 2 weeks after 2nd dose)	90.3 ± 4.3 nmol/L	No change in fasting glucose, postchallenge insulin, Avignon's insulin sensitivity, QUICKI, or HOMA-IR	Not randomized, placebo-controlled; indirect measures of insulin sensitivity
Nyomba et al. 1986 [75]	10 Belgian subjects with epilepsy (mean age: 56 years), and 15 elderly subjects (mean age: 78 years)	17 ± 9.5 nmol/L; 19 ± 19.4 nmol/L	OGTT	Oral, 25(OH)D loading dose of 200 *μ*g + 10 *μ*g/d	2 weeks	36 ± 6 nmol/L 35 ± 3 nmol/L	Decrease in fasting insulin and postchallenge insulin only in subjects with epilepsy	Not randomized, placebo-controlled; small sample size; indirect measure of insulin sensitivity
Lind et al. 1989 [52]	14 normal-weight, Danish men with impaired glucose tolerance, ages 60–63 years	—	IVGTT	Oral, 2 *μ*g/d alphacalcidiol	18 months	78 ± 43 nmol/L	No change in insulin sensitivity	Not randomized, placebo-controlled; baseline 25(OH)D not assessed; small sample size
Ljunghall et al. 1987 [76]	65 vitamin D sufficient, Caucasian men with impaired glucose tolerance, ages 61–65 years, mean BMI: 27.5 kg/m^2^ (treatment), 28.2 kg/m^2^ (placebo)	92.4 ± 23.5 nmol/L (treatment) 97.3 ± 72.4 nmol/L (placebo)	IVGTT	Oral, 0.75 *μ*g/d alphacalcidiol versus placebo	3 months	104.8 ± 20.7 nmol/L (treatment) 134.8 ± 119.8 nmol/L (placebo)	No change in insulin sensitivity	—
Fliser et al. 1997 [77]	18 healthy German males, mean age: 26 years, mean BMI: 22.4 kg/m^2^	—	Euglycemic clamp	Oral, 1.5 *μ*g 1,25(OH)D/d versus placebo	7 days	—	No change in insulin sensitivity	25(OH)D not reported; small sample size

**Table 3:** Associations of VDR polymorphisms with insulin secretion, insulin resistance, and T2DM. VDR: vitamin D receptor; T2DM: type 2 diabetes mellitus; OGTT: oral glucose tolerance test; HDL-C: high density lipoprotein cholesterol; HOMA-IR: homeostasis model assessment of insulin resistance; AUC: area under the curve; WHR, waist-to-hip ratio; HOMA-*β*: homeostasis model assessment of *β*-cell function; LDL-C: low density lipoprotein cholesterol; OR: odds ratio; HbA1c: glycosylated hemoglobin.

Author, year (ref)	Subject Characteristics	VDR gene investigated	Measurement of insulin metabolism	Main outcome	Notes
(a) Insulin Secretion					

Speer et al. [78] 2001	49 Caucasians with T2DM, 29 subjects with obesity but no T2DM, 138 healthy controls	*Bsm*I	Fasting and postprandial C-peptide	Higher postprandial C-peptide in BB genotype among T2DM and obese subjects	Highest postprandial C-peptide in subjects with BB and estrogen receptor polymorphism
Hitman et al. 1998 [79]	171 Bangladeshi Asians	*Taq*I*, Bsm*I*, Apa*I	OGTT	Higher insulin secretion index in AA genotype	No genotype differences in 32, 33 split proinsulin
Ogunkolade et al. 2002 [80]	143 healthy Bangladeshi Asians	*Taq*I*, Bsm*I*, Fok*l*, Apa*I	OGTT	*Taq*I genotype independently predicted insulin secretion index; tt genotypes had greatest insulin secretion index	—

(b) Insulin resistance					

Filus et al. 2008 [81]	176 Polish men	*Fok*l*, Bsm*I	Fasting insulin and glucose	FF/Ff genotype associated with greater fasting insulin than ff	BB genotype associated with greater BMI and waist circumference; FF genotype associated with lower HDL-C
Chiu et al. 2001 [82]	49 glucose-tolerant Caucasian Americans	*Fok*I	Fasting insulin and glucose	ff/Ff genotypes had higher fasting insulin and HOMA-IR than FF; *Fok*I genotype independently predicted HOMA-IR	ff/Ff genotypes had higher postchallenge insulin AUC; higher WHR in ff/Ff genotypes; HOMA-*β* did not differ between genotypes
Oh and Barret-Connor 2002 [83]	1545 Caucasian Americans	*Taq*I*, Bsm*I*, Apa*I	Fasting insulin and glucose	Fasting glucose higher in aa genotype (*Apa*I); HOMA-IR higher in BB genotype (*Bsm*I)	Trend towards higher aa frequency in subjects with T2DM versus those without (*P* = .058)
Ortlepp et al. 2003 [84]	1539 healthy, male German soldiers	*Bsm*I	Fasting glucose	Fasting glucose independently associated with *Bsm*I VDR (BB genotype) only in subjects with low physical activity	—
Tworowska-Bardińska et al. 2008 [85]	350 healthy, Polish postmenopausal women	*Bsm*I	Fasting insulin and glucose	No difference in fasting insulin, glucose, or fasting insulin resistance index between genotypes	Greater LDL-C in BB genotype among those with central obesity

(c) Type 2 Diabetes					

Ortlepp et al. 2003 [84]	293 German adults at risk for coronary artery disease	*Bsm*I	—	Higher T2DM prevalence in BB versus bb genotype; OR of 3.64 (CI: 1.53–8.55)	Higher coronary artery disease prevalence in BB genotype
Dilmec et al. 2009 [86]	72 Turkish adults with T2DM versus 169 healthy controls	*Taq*I*, Apa*I	—	No statistical difference in genotype frequencies among cases versus controls	No difference in fasting plasma glucose or HbA1c among subjects with VDR polymorphisms versus those without
Malecki et al. 2003 [87]	308 Polish adults with T2DM versus 240 controls	*Taq*I*, Bsm*I*, Fok*l*, Apa*I	—	No statistical difference in genotype frequencies among cases versus controls	—
Ye 2001 et al. [88]	309 Caucasians (French) with T2DM versus 143 controls	*Taq*I*, Bsm*I*, Tru*9I*, Apa*I	—	No statistical difference in genotype frequencies among cases versus controls	TT (*Taq*I) and bb (*Bsm*I) genotypes associated with BMI among subjects with early onset T2DM
Boullu-Sanchis et al. 1999 [89]	89 migrant Indians of Guadeloupe with T2DM versus 100 controls	*Taq*I*, Apa*I	—	No statistical difference in genotype frequencies among cases versus controls	—

## References

[1] Heaney RP (2004). Functional indices of vitamin D status and ramifications of vitamin D deficiency. *The American journal of clinical nutrition*.

[2] Holick MF (2007). Medical progress: vitamin D deficiency. *The New England Journal of Medicine*.

[3] Scragg R, Holdaway I, Singh V, Metcalf P, Baker J, Dryson E (1995). Serum 25-hydroxyvitamin D3 levels decreased in impaired glucose tolerance and diabetes mellitus. *Diabetes Research and Clinical Practice*.

[4] Baynes KCR, Boucher BJ, Feskens EJM, Kromhout D (1997). Vitamin D, glucose tolerance anal insulinaemia in elderly men. *Diabetologia*.

[5] Scragg R, Sowers M, Bell C (2004). Serum 25-hydroxyvitamin D, diabetes, and ethnicity in the Third National Health and Nutrition Examination Survey. *Diabetes Care*.

[6] Saintonge S, Bang H, Gerber LM (2009). Implications of a new definition of vitamin D deficiency in a multiracial US adolescent population: the National Health and Nutrition Examination Survey III. *Pediatrics*.

[7] Brancati FL, Kao WHL, Folsom AR, Watson RL, Szklo M (2000). Incident type 2 diabetes mellitus in African American and white adults: the Atherosclerosis Risk in Communities Study. *Journal of the American Medical Association*.

[8] de Souza CJ, Meier AH (1987). Circadian and seasonal variations of plasma insulin and cortisol concentrations in the Syrian hamster, Mesocricetus auratus. *Chronobiology International*.

[9] Pittas AG, Lau J, Hu FB, Dawson-Hughes B (2007). The role of vitamin D and calcium in type 2 diabetes. A systematic review and meta-analysis. *Journal of Clinical Endocrinology and Metabolism*.

[10] Hypponen E, Laara E, Reunanen A, Jarvelin M-R, Virtanen SM (2001). Intake of vitamin D and risk of type 1 diabetes: a birth-cohort study. *The Lancet*.

[11] Prentki M, Nolan CJ (2006). Islet *β* cell failure in type 2 diabetes. *Journal of Clinical Investigation*.

[12] Johnson JA, Grande JP, Roche PC, Kumar R (1994). Immunohistochemical localization of the 1,25(OH)2D3 receptor and calbindin D28k in human and rat pancreas. *American Journal of Physiology*.

[13] Bland R, Markovic D, Hills CE (2004). Expression of 25-hydroxyvitamin D3-1*α*-hydroxylase in pancreatic islets. *Journal of Steroid Biochemistry and Molecular Biology*.

[14] Maestro B, Davila N, Carranza MC, Calle C (2003). Identification of a Vitamin D response element in the human insulin receptor gene promoter. *Journal of Steroid Biochemistry and Molecular Biology*.

[15] Simpson RU, Thomas GA, Arnold AJ (1985). Identification of 1,25-dihydroxyvitamin D3 receptors and activities in muscle. *The Journal of Biological Chemistry*.

[16] Maestro B, Molero S, Bajo S, Dávila N, Calle C (2002). Transcriptional activation of the human insulin receptor gene by 1,25-dihydroxyvitamin D3. *Cell Biochemistry and Function*.

[17] Dunlop TW, Väisänen S, Frank C, Molnár F, Sinkkonen L, Carlberg C, Karn J (2005). The human peroxisome proliferator-activated receptor *δ* gene is a primary target of 1*α*, 25-dihydroxyvitamin D3 and its nuclear receptor. *Journal of Molecular Biology*.

[18] Maestro B, Campion J, Davila N, Calle C (2000). Stimulation by 1,25-dihydroxyvitamin D3 of insulin receptor expression and insulin responsiveness for glucose transport in U-937 human promonocytic cells. *Endocrine Journal*.

[19] Cade C, Norman AW (1986). Vitamin D3 improves impaired glucose tolerance and insulin secretion in the vitamin D-deficient rat in vivo. *Endocrinology*.

[20] Norman AW, Frankel BJ, Heldt AM, Grodsky GM (1980). Vitamin D deficiency inhibits pancreatic secretion of insulin. *Science*.

[21] Clark SA, Stumpf WE, Sar M (1981). Effect of 1,25 dihydroxyvitamin D3 on insulin secretion. *Diabetes*.

[22] Nyomba BL, Bouillon R, De Moor P (1984). Influence of vitamin D status on insulin secretion and glucose tolerance in the rabbit. *Endocrinology*.

[23] Zeitz U, Weber K, Soegiarto DW, Wolf E, Balling R, Erben RG (2003). Impaired insulin secretory capacity in mice lacking a functional vitamin D receptor. *The FASEB Journal*.

[24] Bourlon P-M, Billaudel B, Faure-Dussert A (1999). Influence of vitamin D3 deficiency and 1,25 dihydroxyvitamin D3 on de novo insulin biosynthesis in the islets of the rat endocrine pancreas. *Journal of Endocrinology*.

[25] Zhou QG, Hou FF, Guo ZJ, Liang M, Wang GB, Zhang X (2008). 1,25-dihydroxyvitamin D improved the free fatty-acid-induced insulin resistance in cultured C2C12 cells. *Diabetes/Metabolism Research and Reviews*.

[26] Bischoff HA, Borchers M, Gudat F (2001). In situ detection of 1,25-dihydroxyvitamin D3 receptor in human skeletal muscle tissue. *Histochemical Journal*.

[27] Norman AW (2006). Minireview: vitamin D receptor: new assignments for an already busy receptor. *Endocrinology*.

[28] Li J, Byrne ME, Chang E (2008). 1*α*,25-dihydroxyvitamin D hydroxylase in adipocytes. *Journal of Steroid Biochemistry and Molecular Biology*.

[29] Bischoff-Ferrari HA, Borchers M, Gudat F,  Durmuller U, Stahelin HB, Dick W (2004). Vitamin D receptor expression in human muscle tissue decreases with age. *Journal of Bone and Mineral Research*.

[30] Draznin B (1993). Cytosolic calcium and insulin resistance. *American Journal of Kidney Diseases*.

[31] Reusch JE-B, Begum N, Sussman KE, Draznin B (1991). Regulation of GLUT-4 phosphorylation by intracellular calcium in adipocytes. *Endocrinology*.

[32] Worrall DS, Olefsky JM (2002). The effects of intracellular calcium depletion on insulin signaling in 3T3-L1 adipocytes. *Molecular Endocrinology*.

[33] Wollheim CB, Sharp GW (1981). Regulation of insulin release by calcium. *Physiological Reviews*.

[34] Bjorklund A, Lansner A, Grill VE (2000). Glucose-induced [Ca^2+^](i) abnormalities in human pancreatic islets: important role of overstimulation. *Diabetes*.

[35] Zemel MB (1998). Nutritional and endocrine modulation of intracellular calcium: implications in obesity, insulin resistance and hypertension. *Molecular and Cellular Biochemistry*.

[36] Munshi HG, Burks DJ, Joyal JL, White MF, Sacks DB (1996). Ca^2+^ regulates calmodulin binding to IQ motifs in IRS-1. *Biochemistry*.

[37] Li Z, Joyal JL, Sacks DB (2000). Binding of IRS proteins to calmodulin is enhanced in insulin resistance. *Biochemistry*.

[38] McCarty MF, Thomas CA (2003). PTH excess may promote weight gain by impeding catecholamine-induced lipolysis-implications for the impact of calcium, vitamin D, and alcohol on body weight. *Medical Hypotheses*.

[39] Chiu KC, Chuang L-M, Lee NP (2000). Insulin sensitivity is inversely correlated with plasma intact parathyroid hormone level. *Metabolism*.

[40] Kamycheva E, Jorde R, Figenschau Y, Haug E (2007). Insulin sensitivity in subjects with secondary hyperparathyroidism and the effect of a low serum 25-hydroxyvitamin D level on insulin sensitivity. *Journal of Endocrinological Investigation*.

[41] Boucher BJ, Mannan N, Noonan K, Hales CN, Evans SJW (1995). Glucose intolerance and impairment of insulin secretion in relation to vitamin D deficiency in East London Asians. *Diabetologia*.

[42] Orwoll E, Riddle M, Prince M (1994). Effects of vitamin D on insulin and glucagon secretion in non-insulin-dependent diabetes mellitus. *American Journal of Clinical Nutrition*.

[43] Chiu KC, Chu A, Go VLW, Saad MF (2004). Hypovitaminosis D is associated with insulin resistance and *β* cell dysfunction. *American Journal of Clinical Nutrition*.

[44] Reaven GM (1988). Role of insulin resistance in human disease. *Diabetes*.

[45] Chonchol M, Scragg R (2007). 25-hydroxyvitamin D, insulin resistance, and kidney function in the Third National Health and Nutrition Examination Survey. *Kidney International*.

[46] McGill A-T, Stewart JM, Lithander FE, Strik CM, Poppitt SD (2008). Relationships of low serum vitamin D3 with anthropometry and markers of the metabolic syndrome and diabetes in overweight and obesity. *Nutrition Journal*.

[47] Gannage-Yared MH, Chedid R, Khalife S, Azzi E, Zoghbi F, Halaby G (2009). Vitamin D in relation to metabolic risk factors, insulin sensitivity and adiponectin in a young Middle-Eastern population. *European Journal of Endocrinology*.

[48] Lu L, Yu Z, Pan A (2009). Plasma 25-hydroxyvitamin D concentration and metabolic syndrome among middle-aged and elderly Chinese individuals. *Diabetes Care*.

[49] Clifton-Bligh RJ, McElduff P, McElduff A (2008). Maternal vitamin D deficiency, ethnicity and gestational diabetes. *Diabetic Medicine*.

[50] Alemzadeh R, Kichler J, Babar G, Calhoun M (2008). Hypovitaminosis D in obese children and adolescents: relationship with adiposity, insulin sensitivity, ethnicity, and season. *Metabolism*.

[51] Lind L, Hanni A, Lithell H, Hvarfner A, Sorensen OH, Ljunghall S (1995). Vitamin D is related to blood pressure and other cardiovascular risk factors in middle-aged men. *American Journal of Hypertension*.

[52] Lind L, Pollare T, Hvarfner A, Lithell H, Sorensen OH, Ljunghall S (1989). Long-term treatment with active vitamin D (alphacalcidol) in middle-aged men with impaired glucose tolerance. Effects on insulin secretion and sensitivity, glucose tolerance and blood pressure. *Diabetes Research*.

[53] Ford ES, Ajani UA, McGuire LC, Liu S (2005). Concentrations of serum vitamin D and the metabolic syndrome among U.S. adults. *Diabetes Care*.

[54] Kim SH, Abbasi F, Reaven GM (2004). Impact of degree of obesity on surrogate estimates of insulin resistance. *Diabetes Care*.

[55] Reis JP, von Muhlen D, Kritz-Silverstein D, Wingard DL, Barrett-Connor E (2007). Vitamin D, parathyroid hormone levels, and the prevalence of metabolic syndrome in community-dwelling older adults. *Diabetes Care*.

[56] McKinney K, Breitkopf CR, Berenson AB (2008). Association of race, body fat and season with vitamin D status among young women: a cross-sectional study. *Clinical Endocrinology*.

[57] Snijder MB, van Dam RM, Visser M (2005). Adiposity in relation to vitamin D status and parathyroid hormone levels: a population-based study in older men and women. *Journal of Clinical Endocrinology and Metabolism*.

[58] Arunabh S, Pollack S, Yeh J, Aloia JF (2003). Body fat content and 25-hydroxyvitamin D levels in healthy women. *Journal of Clinical Endocrinology and Metabolism*.

[59] Manco M, Calvani M, Nanni G (2005). Low 25-hydroxyvitamin d does not affect insulin sensitivity in obesity after bariatric surgery. *Obesity Research*.

[60] Mattila C, Knekt P, Mannisto S (2007). Serum 25-hydroxyvitamin D concentration and subsequent risk of type 2 diabetes. *Diabetes Care*.

[61] Forouhi NG, Luan J, Cooper A, Boucher BJ, Wareham NJ (2008). Baseline serum 25-hydroxy vitamin d is predictive of future glycemic status and insulin resistance the medical research council ely prospective study 1990–2000. *Diabetes*.

[62] Knekt P, Laaksonen M, Mattila C (2008). Serum vitamin D and subsequent occurrence of type 2 diabetes. *Epidemiology*.

[63] Need AG, O'Loughlin PD, Horowitz M, Nordin BEC (2005). Relationship between fasting serum glucose, age, body mass index and serum 25 hydroxyvitamin D in postmenopausal women. *Clinical Endocrinology*.

[64] Hypponen E, Boucher BJ, Berry DJ, Power C (2008). 25-hydroxyvitamin D, IGF-1, and metabolic syndrome at 45 years of age: a cross-sectional study in the 1958 British Birth Cohort. *Diabetes*.

[65] Gedik O, Akalin S (1986). Effects of vitamin D deficiency and repletion on insulin and glucagon secretion in man. *Diabetologia*.

[66] Borissova A-M, Tankova T, Kirilov G, Dakovska L, Kovacheva R (2003). The effect of vitamin D3 on insulin secretion and peripheral insulin sensitivity in type 2 diabetic patients. *International Journal of Clinical Practice*.

[67] Inomata S, Kadowaki S, Yamatani T (1986). Effect of 1*α*(OH)-vitamin D3 on insulin secretion in diabetes mellitus. *Bone and Mineral*.

[68] Zofkova I, Stolba P (1990). Effect of calcitriol and trifluoperazine on glucose stimulated B cell function in healthy humans. *Experimental and Clinical Endocrinology*.

[69] Jorde R, Figenschau Y (2009). Supplementation with cholecalciferol does not improve glycaemic control in diabetic subjects with normal serum 25-hydroxyvitamin D levels. *European Journal of Nutrition*.

[70] Nagpal J, Pande JN, Bhartia A (2009). A double-blind, randomized, placebo-controlled trial of the short-term effect of vitamin D3 supplementation on insulin sensitivity in apparently healthy, middle-aged, centrally obese men. *Diabetic Medicine*.

[71] de Boer IH, Tinker LF, Connelly S (2008). Calcium plus vitamin D supplementation and the risk of incident diabetes in the women's health initiative. *Diabetes Care*.

[72] Nilas L, Christiansen C (1984). Treatment with vitamin D or its analogues does not change body weight or blood glucose level in postmenopausal women. *International Journal of Obesity*.

[73] Pittas AG, Harris SS, Stark PC, Dawson-Hughes B (2007). The effects of calcium and vitamin D supplementation on blood glucose and markers of inflammation in nondiabetic adults. *Diabetes Care*.

[74] Tai K, Need AG, Horowitz M, Chapman IM (2008). Glucose tolerance and vitamin D: effects of treating vitamin D deficiency. *Nutrition*.

[75] Nyomba BL, Auwerx J, Bormans V (1986). Pancreatic secretion in man with subclinical vitamin D deficiency. *Diabetologia*.

[76] Ljunghall S, Lind L, Lithell H (1987). Treatment with one-alpha-hydroxycholecalciferol in middle-aged men with impaired glucose tolerance. A prospective randomized double-blind study. *Acta Medica Scandinavica*.

[77] Fliser D, Stefanski A, Franek E, Fode P, Gudarzi A, Ritz E (1997). No effect of calcitriol on insulin-mediated glucose uptake in healthy subjects. *European Journal of Clinical Investigation*.

[78] Speer G, Cseh K, Winkler G (2001). Vitamin D and estrogen receptor gene polymorphisms in type 2 diabetes mellitus and in android type obesity. *European Journal of Endocrinology*.

[79] Hitman GA, Mannan N, McDermott MF (1998). Vitamin D receptor gene polymorphisms influence insulin secretion in Bangladeshi Asians. *Diabetes*.

[80] Ogunkolade B-W, Boucher BJ, Prahl JM (2002). Vitamin D receptor (VDR) mRNA and VDR protein levels in relation to vitamin D status, insulin secretory capacity, and VDR genotype in Bangladeshi Asians. *Diabetes*.

[81] Filus A, Trzmiel A, Kuliczkowska-Płaksej J (2008). Relationship between vitamin D receptor BsmI and FokI polymorphisms and anthropometric and biochemical parameters describing metabolic syndrome. *Aging Male*.

[82] Chiu KC, Chuang L-M, Yoon C (2001). The vitamin D receptor polymorphism in the translation initiation codon is a risk factor for insulin resistance in glucose tolerant Caucasians. *BMC Medical Genetics*.

[83] Oh J-Y, Barrett-Connor E (2002). Association between vitamin D receptor polymorphism and type 2 diabetes or metabolic syndrome in community-dwelling older adults: the Rancho Bernardo Study. *Metabolism*.

[84] Ortlepp JR, Metrikat J, Albrecht M, von Korff A, Hanrath P, Hoffmann R (2003). The vitamin D receptor gene variant and physical activity predicts fasting glucose levels in healthy young men. *Diabetic Medicine*.

[85] Tworowska-Bardzinska U, Lwow F, Kubicka E (2008). The vitamin D receptor gene BsmI polymorphism is not associated with anthropometric and biochemical parameters describing metabolic syndrome in postmenopausal women. *Gynecological Endocrinology*.

[86] Dilmec F, Uzer E, Akkafa F, Kose E, van Kuilenburg AB Detection of *VDR* gene *ApaI* and *TaqI* polymorphisms in patients with type 2 diabetes mellitus using PCR-RFLP method in a Turkish population.

[87] Malecki MT, Frey J, Moczulski D, Klupa T, Kozek E, Sieradzki J (2003). Vitamin D receptor gene polymorphisms and association with type 2 diabetes mellitus in a Polish population. *Experimental and Clinical Endocrinology and Diabetes*.

[88] Ye W-Z, Reis AF, Dubois-Laforgue D, Bellanne-Chantelot C, Timsit J, Velho G (2001). Vitamin D receptor gene polymorphisms are associated with obesity in type 2 diabetic subjects with early age of onset. *European Journal of Endocrinology*.

[89] Boullu-Sanchis S, Lepretre F, Hedelin G (1999). Type 2 diabetes mellitus: association study of five candidate genes in an Indian population of Guadeloupe, genetic contribution of FABP2 polymorphism. *Diabetes and Metabolism*.

[90] Holick MF (2004). Vitamin D: importance in the prevention of cancers, type 1 diabetes, heart disease, and osteoporosis. *American Journal of Clinical Nutrition*.

[91] Dusso AS, Brown AJ, Slatopolsky E (2005). Vitamin D. *American Journal of Physiology*.

[92] Gunal AI, Celiker H, Celebi H, Ustundag B, Gunal SY (1997). Intravenous alfacalcidol improves insulin resistance in hemodialysis patients. *Clinical Nephrology*.

[93] Kautzky-Willer A, Pacini G, Barnas U (1995). Intravenous calcitriol normalizes insulin sensitivity in uremic patients. *Kidney International*.

[94] Pepper KJ, Judd SE, Nanes MS, Tangpricha V (2009). Evaluation of vitamin d repletion regimens to correct vitamin d status in adults. *Endocrine Practice*.

[95] Gómez JM (2006). The role of insulin-like growth factor I components in the regulation of vitamin D. *Current Pharmaceutical Biotechnology*.

[96] Bianda T, Glatz Y, Bouillon R, Froesch ER, Schmid C (1998). Effects of short-term insulin-like growth factor-I (IGF-I) or growth hormone (GH) treatment on bone metabolism and on production of 1,25- dihydroxycholecalciferol in GH-deficient adults. *Journal of Clinical Endocrinology and Metabolism*.

